# Bovine colostrum supplementation as a new perspective in depression and substance use disorder treatment: a randomized placebo-controlled study

**DOI:** 10.3389/fpsyt.2024.1366942

**Published:** 2024-06-18

**Authors:** Krzysztof Durkalec-Michalski, Natalia Główka, Tomasz Podgórski, Weronika Odrobny, Marcin Krawczyński, Ryszard Botwina, Stanisław Bodzicz, Paulina M. Nowaczyk

**Affiliations:** ^1^ Department of Sports Dietetics, Poznań University of Physical Education, Poznań, Poland; ^2^ Sport Sciences–Biomedical Department, Charles University, Prague, Czechia; ^3^ Department of Physiology and Biochemistry, Poznań University of Physical Education, Poznań, Poland; ^4^ Institute of Mental Health Para Familia, Gorzów Wielkopolski, Poland; ^5^ Faculty of Physical Education, Gdansk University of Physical Education and Sport, Gdańsk, Poland; ^6^ Agrapak Company, Poznań, Poland

**Keywords:** substance use disorder, major depressive disorder, nutrition, add-on treatment, blood markers, inflammation, cytokine

## Abstract

**Introduction:**

This randomized, placebo-controlled, double-blind, parallel study aimed to evaluate the effect of 3-month supplementation of bovine colostrum (BOV-COL; 8*x*400 mg per day) on the outcomes of depression treatment in hospitalized patients with substance use disorder (SUD). The hypothesis is that BOV-COL supplementation as an add-on treatment results in favorable alternations in selected blood inflammatory markers or neurotransmitters, leading to better depression treatment outcomes compared with placebo (PLA).

**Methods:**

Patients with a Minnesota Multiphasic Personality Inventory-2 score ≥60 points were enrolled. Twenty-nine participants (*n*=18 in the BOV-COL group and *n*=11 in the PLA group) completed the protocol.

**Results:**

The mean Beck Depression Inventory-II score was significantly reduced after supplementation in both groups. However, the mean 17-point Hamilton Depression Rating Scale score was decreased in the BOV-COL group, but not in the PLA group. In the BOV-COL group, there was a reduction in interleukin (IL)-1, IL-6, IL-10, the IL-6:IL-10 ratio, IL-17, and tumor necrosis factor alpha (TNF-α), while in the PLA group only IL-6 decreased. Favorable alternations in the total count and differentials of white blood cell subsets were more pronounced in the BOV-COL. There were no changes in neurotransmitter concentrations.

**Conclusions:**

BOV-COL supplementation is a promising add-on therapy in patients with depression and SUD.

## 1 Introduction

Depression can be categorized as a mood disturbance that may last for more than 2 weeks in a row. Nearly 265 million people throughout the world experience depression, of which more than 700 000 commit suicide every year (1). Among the factors that influence the occurrence of major depressive disorder (MDD), in addition to child abuse, neglect, loss, or bad life experiences, there are biological and genetic conditions, as well as other still unclear/unrevealed causes (2, 3). Moreover, MDD is the most common co-occurring psychiatric disorder among people with alcohol or substance use disorders (SUD) (4), as diagnosed based on the Diagnostic and Statistical Manual of Mental Disorders (5th ed., DSM-V) (5, 6). According to McHugh and Weiss (5), people with diagnosed alcohol dependence are 3.7 times more likely to have MDD, and 2.8 times more likely to have a history of dysthymia.

Interestingly, low-grade inflammation represents a potential pathophysiological mechanism of depression (7). The literature supports the view that the immune system and inflammatory processes may contribute to psychiatric disorders. Stress, as one of the factors, induces activation of the immune response and alters neurotransmission, leading to neurotransmitter imbalances (8, 9). Pro-inflammatory cytokines are important triggers for depression and may integrate the endocrine and neurotransmitter changes with the impact of inflammation on the neuron (9). An increase in the inflammatory markers, like C-reactive protein (CRP), interleukin (IL)-6, and tumor necrosis factor alpha (TNF-α), is often observed in patients with depression compared with healthy individuals. Recently, the new field of “psychoimmunology” has emphasized the importance of alterations in inflammatory markers on neurodegenerative symptoms (7). In their meta-analysis, Więdłocha et al. (10) found a decrease in inflammatory markers (CRP, IL-4, IL-6, IL-10, IL-1β, TNF-α, and C-C motif chemokine ligand-2 [CCL-2]) during short-term antidepressant treatment. Nevertheless, there are few studies that have investigated the correlations between inflammatory markers and long-term depression outcomes. For example, higher levels of IL-6 (11) and CRP (12) have been associated with an increased risk of the subsequent depression. On the other hand, inflammation is present in only a subgroup of patients with depression (13).

Moreover, depression has been linked to altered neuronal integrity and abnormal functional activity in different brain areas. Several neurotransmitters like dopamine (DOP), epinephrine, norepinephrine, gamma-aminobutyric acid (GABA), and glutamate may affect mood. Their complex interactions with receptors may affect the brain’s chemical moods and play a role in depressive disorders (14).

Different types of depression can be effectively treated with five main groups of antidepressants (15–17). However, meta-analyses suggest that there are low remission rates and they provide clinically relevant outcomes only in patients with upper-end severe depression (18). On the other hand, several nutrients and food constituents have been evaluated regarding a decrease in depression symptoms, for example, probiotics and prebiotics, polyunsaturated fatty acids (PUFA), vitamin D, vitamin B12, magnesium, zinc, and curcumin (19). Bovine colostrum (BOV-COL) also seems to have potential in this regard. This substance is produced naturally by the mammary glands of cows for 24–72 hours after calving. The biologically active ingredients of BOV-COL fall into three main categories: nutrients, immune factors, and growth factors. Although the final composition of BOV-COL may vary to some extent depending on the time of the milking, environmental conditions, and the actual needs of the newborn calf, the available data indicate its high nutritional value (20). It contains about 100-fold higher concentrations of immunoglobulins (Ig) and higher concentrations of lactoferrin than mature milk (20–23). BOV-COL is assumed to be one of the strongest natural immune stimulants (24). BOV-COL ingestion has a substantial impact on the proper functioning of immune system of calves, thus it is assumed that the use of BOV-COL-based products may be also beneficial in humans (20, 21, 23). Evidence suggests that BOV-COL may have numerous multidirectional clinical or therapeutic applications in humans (20). The ingestion of BOV-COL can modulate the function of subsets of lymphocytes, macrophages, and dendritic cells, and increase regulatory cytokines such as IL-10 (25). The main growth factor in BOV-COL is insulin-like growth factor-1 (IGF-1), which modulates, inter alia, stimulation of growth maintenance and the functioning of the muscle tissue (26). BOV-COL supplementation is considered to be well tolerated and safe for the adult human. The reported adverse effects include only mild complaints such as nausea, diarrhea, flatulence, an unpleasant taste, and abdominal discomfort, which may disappear with time. Still, there are no existing data for long-term (≥3 months) use of BOV-COL; therefore, no conclusions can be currently made (20, 27).

Finally, although BOV-COL is a potentially anti-inflammatory substance, there are no data on the efficacy of BOV-COL supplementation in patients with depression. Therefore, we aimed to evaluate the effect of long-term BOV-COL supplementation on ratings of depression in patients with SUD undergoing therapy in the addiction rehabilitation center and diagnosed with co-occurring clinically recognized depression. We hypothesize that the implementation of BOV-COL supplementation as an adjunct to currently ongoing depression treatment results in favorable alternations in selected inflammatory and hematological markers and/or neurotransmitters, leading to better depression treatment outcomes compared with placebo (PLA) supplementation. This eventuality would suggest that BOV-COL is a potential natural substance that can enhance therapy outcomes in individuals with depression.

## 2 Materials and methods

### 2.1 Study participants

**Figure 1:**
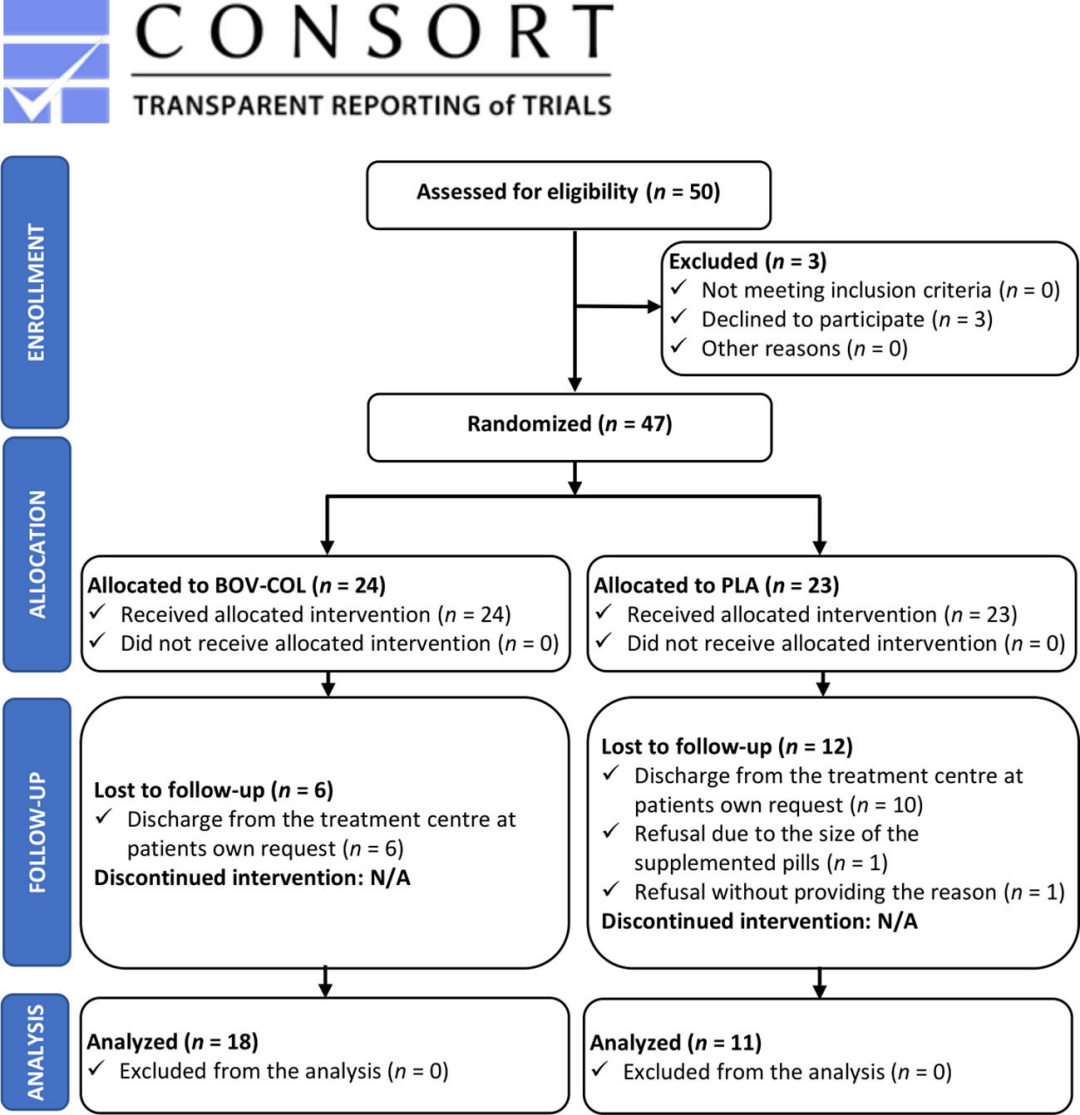
Flowchart of the study design.

The study participants were enrolled based on the Minnesota Multiphasic Personality Inventory-2 (MMPI-2) (28–30). A score corresponding to elevated depression symptoms (i.e., ≥60 points in depression clinical scale) was established as an inclusion criterion (**Table 1**). Initially, the study group consisted of 47 patients undergoing therapy in the addiction rehabilitation center (located in Lubuskie voivodeship, west Poland) and with clinically diagnosed depression at the time of the enrollment (**Figure 1**). All patients were diagnosed with dependence syndrome due to multiple drug use and use of other psychoactive substances [F19.2 according to ICD-10 (31)]. The size of the enrolled sample was conditioned by the number of patients treated in the center who met the inclusion criteria and were willing to participate in the protocol. The baseline assumptions was to conduct the investigation as a single-center study. The patients were randomized to the BOV-COL or PLA group via block randomization using an online randomizer tool (www.sealedenvelope.com; block size: 2; actual list length: 50). There were 18 dropouts from the study protocol. The main reasons for dropping out were: discharge from the treatment center at the patient’s request (*n* = 16), refusal to participate due to the size of the supplemented pills (*n* = 1), and refusal without providing the reason (*n* = 1). The baseline characteristics of study participants according to full completion of the entire study protocol (*n* = 29) or dropping out from the study (*n* = 18) is provided in the **Supplementary Table 1**. Finally, the study group and statistical analyses covered 29 patients (5 women and 24 men; **Table 1**).

**Table 1:** Basic characteristics of the final studied groups.

		Unit	BOV-COL	PLA	*p*
		(*n*)	18	11	
Women/men	–	(*n*)	4/14	1/10	–
Age	–	(years)	28.0 ± 7.6	32.7 ± 8.6	0.135^1^
Body mass	–	(kg)	76.9 ± 15.8	83.2 ± 11.0	0.284^1^
Body height	–	(cm)	177 ± 12	177 ± 5	0.993^1^
BMI	–	(kg·(m^2^)^-1^)	24.3 ± 3.5	26.5 ± 3.5	0.140^1^
MMPI-2	–	(points)	70 ± 7	76 ± 8	0.038^1^
MMPI-2 classification	Elevated	(*n*/%)	4/22	1/9	0.219^2^
	High	(*n*/%)	11/61	5/45.5	*χ^2^ = *3.037
	Very high	(*n*/%)	3/17	5/45.5	
Duration of the stay at the treatment center at the start of the supplementation	–	(months)	5 ± 3	6 ± 4	0.803^1^

BMI, body mass index; MMPI-2, Minnesota Multiphasic Personality Inventory-2. ^1^Data analyzed with the *T*-test for independent variables. ^2^Data were analyzed with the chi-square test of independence (and results expressed as Pearson χ^2^).

The time of stay at the addiction rehabilitation center was 5 ± 3 and 6 ± 4 months in the BOV-COL and PLA groups, respectively (**Table 1**). The participation in the study did not disrupt the individual courses of the patients’ treatments and was solely an adjunct to the hitherto implemented treatment, including pharmacotherapy.

All patients declared voluntary willingness to participate in the research. The study protocol was reviewed and approved by the Regional Medical Chamber in Gdansk (reference number KB-39/18, issued on December 20, 2018). All study participants gave written informed consent for participation. All procedures were carried out in accordance with the ethical standards of the Declaration of Helsinki (2013 version).

### 2.2 Study design

The study was a randomized, double-blind, PLA-controlled, parallel-group trial and comprised 3 months of supplementation with either BOV-COL or PLA in a group of patients with clinically diagnosed depression (**Figure 1**). All participants started and completed the study protocol on the same dates; the study was conducted between April and July 2022.

The primary outcome, namely the severity of depression symptoms, was assessed before the start of the supplementation (PRE) and after 3 months of supplementation (POST) by using the Beck Depression Inventory-II (BDI-II) (32–34) and the 17-point Hamilton Depression Rating Scale (HDRS-17) (35). The secondary outcomes were blood concentrations of clinically relevant inflammatory and hematological markers, as well as selected neurotransmitters.

#### 2.2.1 Supplementation

The study participants were supplemented with either BOV-COL or PLA for 3 months. The study was double-blind; thus, the researchers and the study participants were not aware about the received supplementation. The result assessors were not aware of the intervention received by each study participant. Randomization details were anonymized and revealed after protocol cessation. The randomization process was done by a staff member who did not directly participate in the investigations. BOV-COL (Agrapak, Poland) was provided in the form of slow-release gastro-resistant pills. Each pill provided 400 mg of BOV-COL (40% IgG concentration). Skimmed cow milk in powdered form was utilized as a PLA and was provided in the same pill form as BOV-COL. Each participant consumed eight pills in total of the prescribed preparation daily (8 *x* 400 mg = 3200 mg of BOV-COL or PLA daily), split into two doses per day, namely four pills after breakfast and four pills after supper. Compliance with BOV-COL and PLA ingestion was monitored by caregivers at the rehabilitation center.

#### 2.2.2 Depression diagnosis and depression severity evaluation

For the diagnosis of depression, Polish normalized version of the MMPI-2 (28–30) was applied. The tool enables diagnosis of the patient’s functioning in more than 100 dimensions, assessed by: 10 clinical scales – including a scale of depression, 31 clinical subscales, 9 restructured clinical scales, the Personality Psychopathology Five scales, content scales, 27 content component scales, and supplemental scales (28). For the current study, ≥60 points for the scale of depression was established as a qualification criterion.

For the assessment of the severity of depression symptoms and the efficacy of the treatment, the following standardized and validated tools were applied: the BDI-II (32–34, 36) and the HDRS-17 (35, 36).

Based on the BDI-II results, the following classification of depression was applied: no depression (≤13 points), mild depression (14–19 points), moderate depression (20–28 points), and severe depression (29–63 points) (36). Afterwards, the PRE-POST absolute change in the BDI-II score ranging from -1 to -9 points was considered a slight change, between -10 and -19 points as a moderate change, and ≥-20 points as a large change (36–38). Based on the literature (36–38), a 5-point decrease is considered to be a minimally important clinical difference—that is, a smaller difference is enough when the baseline depression is mild, and a larger difference is required when the baseline depression is severe. Any increase in the BDI-II score at the POST evaluation compared with the PRE evaluation that resulted in the qualification of a patient to the group of more intense depressive symptoms was considered to be a worsening of depression severity (i.e., a PRE vs. POST change from 8 points [no depression] to 22 points [moderate depression] was considered to be worsening, while an increase from 6 points [no depression] to 8 points [no depression] was considered to be no change).

Based on the HDRS-17 results, the following classification of depression was applied: no depression (<8 points), mild depression (8–13 points), moderate depression (14–19 points), severe depression (20–25 points), and very severe depression (>25 points) (36). Afterwards, the following percentage ranges for the HDRS-17 score changes were applied to interpret the degree of changes: from -78.2% to -59.1%, very much improved; from -59.0% to -27.2%, much improved; from -27.1% to -6.1%, minimally improved; from -6.0% to -2.1%, no change; from -2.0% to +11.5%, minimally worse; from +11.6% to +20.9%, much worse; and ≥+21.0%, very much worse (36, 39).

#### 2.2.3 Blood collection and sample analysis

Ten milliliters of blood was collected from the ulnar vein early in the morning in a fasted state and centrifuged at 1500 g at 4°C for 5 min (Universal 320R; Hettich Lab Technology, Tuttlingen, Germany) to obtain serum for biochemical analysis. Blood serum was stored at -80°C until biochemical analyses were performed. All inflammatory markers (IL-1, sensitivity [Sens] <10 pg·mL^-1^; IL-4, Sens <7.8 pg·mL^-1^; IL-6, Sens <9.8 pg·mL^-1^; IL-10, Sens <10 pg·mL^-1^; IL-17, Sens <8.9 pg·mL^-1^; IL-21, Sens <7.8 pg·mL^-1^; TNF-α, Sens <3.9 pg·mL^-1^) and neurotransmitters (serotonin [SER], Sens <0.35 ng·mL^-1^ and DOP, Sens <0.23 ng·mL^-1^) were determined by immunoenzymatic ELISA (Wuhan EIAab Science, China). A multi-mode microplate reader (Synergy 2 SIAFRT, BioTek, Winooski, VT, USA) was used for the spectrometric measurements.

Additionally, 1 mL of the remaining venous blood was collected in test tubes containing EDTA dipotassium salt as an anticoagulant for hematological measurements using a 20-parametric automated Mythic^®^ 18 hematology analyzer (Orphée, Geneva, Switzerland). The following hematological indices were immediately evaluated: white blood cells (WBC), lymphocytes (LYM; counts and percentage), monocytes (MON; counts and percentage), granulocytes (GRA; counts and percentage), red blood cells (RBC), hematocrit (HCT), hemoglobin (HGB), mean corpuscular volume of RBC (MCV), mean corpuscular hemoglobin mass in RBC (MCH), mean corpuscular hemoglobin concentration in RBC (MCHC), RBC distribution width – coefficient of variation (RDW-C), RBC distribution width – standard deviation (RDW-S), platelet count (PLT), mean PLT volume (MPV), platelet hematocrit (PCT), PLT distribution width (PDW) and PLT large cell ratio (PLCR). In addition, to avoid potential misinterpretation of blood markers’ results, due to inter-individual variation in hydration status and hematology indices related to the number of blood cellular components (WBC, RBC, HGB, PLT) between study visits all blood biochemical parameters (apart those expressed as percentages or indexes) were converted using previously described hematocrit correction formula (40–43).

#### 2.2.4 Statistical analysis

All variables were checked for a normal distribution with the Shapiro–Wilk test. The results are presented as the mean ± standard deviation (SD) and 95% confidence interval (CI). Between-group comparisons (BOV-COL vs. PLA) were determined with the *T*-test for independent variables, with the effect size expressed as Cohen’s *d* (data with a normal distribution), or the Mann–Whitney *U*-test, with effect size expressed as Glass’s rank-biserial correlation coefficient (*r_g_*) (data with a non-normal distribution). PRE vs. POST comparisons were made with one-way analysis of variance (ANOVA) with repeated measurements, with the effect size expressed as partial eta-squared (*η* ^2^_p_) (data with a normal distribution), or the Wilcoxon signed-rank test, with the effect size expressed as the rank correlation coefficient (*r_c_*) (data with a non-normal distribution). The relationships between inflammatory markers, neurotransmitters, the BDI-II score, and the HDRS-17 score were analyzed with the Pearson correlation coefficient or Spearman’s rank correlation, depending on the data distribution. The chi-square test of independence with Pearson *χ^2^* was used to evaluate classification frequencies (i.e., frequencies between the BOV-COL and PLA groups at the same time point). Statistical significance was set at *p* < 0.05. Moreover, the intention-to-treat (ITT) analysis using linear interpolation was performed for BDI-II and HDRS-17 results. The data were analyzed by using the STATISTICA 13.3 software (StatSoft Inc., Tulsa, OK, USA).

## 3 Results

### 3.1 Depression scales

**Figure 2:**
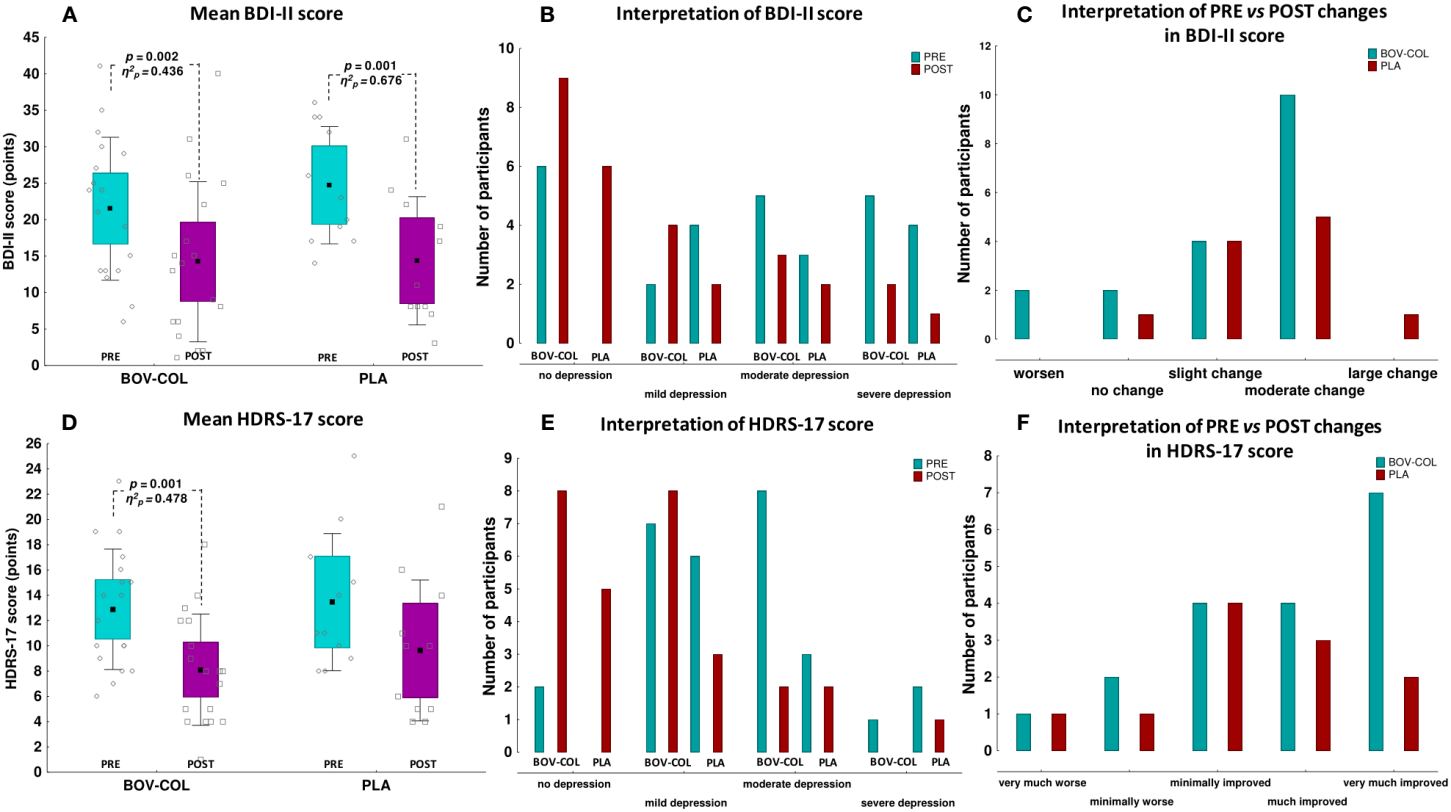
The mean **(A)** Beck Depression Inventory-II (BDI-II) and **(D)** 17-point Hamilton Depression Rating Scale (HDRS-17) scores; classification of depression severity according to the **(B)** BDI-II and **(E)** HDRS-17; and interpretation of PRE vs. POST changes in the **(C)** BDI-II and **(F)** HDRS-17. For **(A, D)**, the data are expressed as the mean (square), 95% confidence interval (box), 95% CI + one standard deviations (whisker), and raw data; the data were analyzed with one-way analysis of variance with repeated measurements (PRE vs. POST comparisons) or the *T*-test for independent variables (BOV-COL vs. PLA comparisons; no differences between the groups at any time point). For **(B–F)**, the data are expressed as the number of participants; the data were analyzed with the chi-square test of independence and Pearson *χ^2^* (BOV-COL vs. PLA comparisons; no differences between the groups at any time point).

There were no differences in the average BDI-II and HDRS-17 scores between the BOV-COL and PLA groups at any time point (**Figures 2A, D**). After the treatment, the BDI-II score was significantly lower in the BOV-COL (*p* = 0.002; *η* ^2^ *_p_* = 0.436) and PLA (*p* = 0.001; *η* ^2^ *_p_* = 0.676; **Figure 2A**) groups. The number of participants categorized into particular classes of depression severity based on the BDI-II score did not differ between the BOV-COL and PLA groups at any time point (**Figure 2B**). Based on PRE-POST changes in the BDI-II score in the BOV-COL group, we observed the following depression severity: *worsening* in 2 patients (11% of the BOV-COL group), *no change* in 2 patients (11%), a *slight change* in 4 patients (22%), and a *moderate change* in 10 patients (56%). In the PLA group, the corresponding number of patients was 0, 1 (9% of the PLA group), 4 (36%), and 5 (46%), respectively, while 1 patient (9%) experienced a *large change* in the depression outcomes (improvement; **Figure 2C**). The BOV-COL and PLA groups did not differ significantly in the outcomes of depression treatment based on the BDI-II changes.

Simultaneously, the HDRS-17 score at the POST evaluation was substantially lower compared with the PRE evaluation in the BOV-COL group (*p* = 0.001; *η* ^2^ *_p_* = 0.478), but not in the PLA group (*p* = 0.071; *η* ^2^ *_p_* = 0.291) (**Figure 2D**). The number of participants categorized to particular classes of depression severity based on the HDRS-17 did not differ between the BOV-COL and PLA groups at any time point (**Figure 2E**). Based on the PRE-POST percentage changes in the HDRS-17 in the BOV-COL group, we observed the following depression severity: *very much worse* in 1 patient (5% of the BOV-COL group), *minimally worse* in 2 patients (11%), *minimally improved* in 4 patients (22%), *much improved* in 4 patients (22%), and *very much improved* in 7 patients (39%) (**Figure 2F**). In the PLA group, the corresponding number of patients was 1 (9% of the PLA group), 1 (9%), 4 (36%), 3 (27%), and 2 (18%), respectively. However, the BOV-COL and PLA groups did not differ significantly in the outcomes of depression treatment based on the HDRS-17 changes.

According to ITT analysis using linear interpolation, the mean BDI-II and HDRS-17 scores were significantly decreased after supplementation in both groups. Still there were no differences in the distributions of *(a)* classes of depression severity or *(b)* classes of changes in depression severity based on BDI-II and HDRS-17 between the BOV-COL and PLA groups (**Supplementary Table 1**).

### 3.2 Inflammatory markers

**Figure 3:**
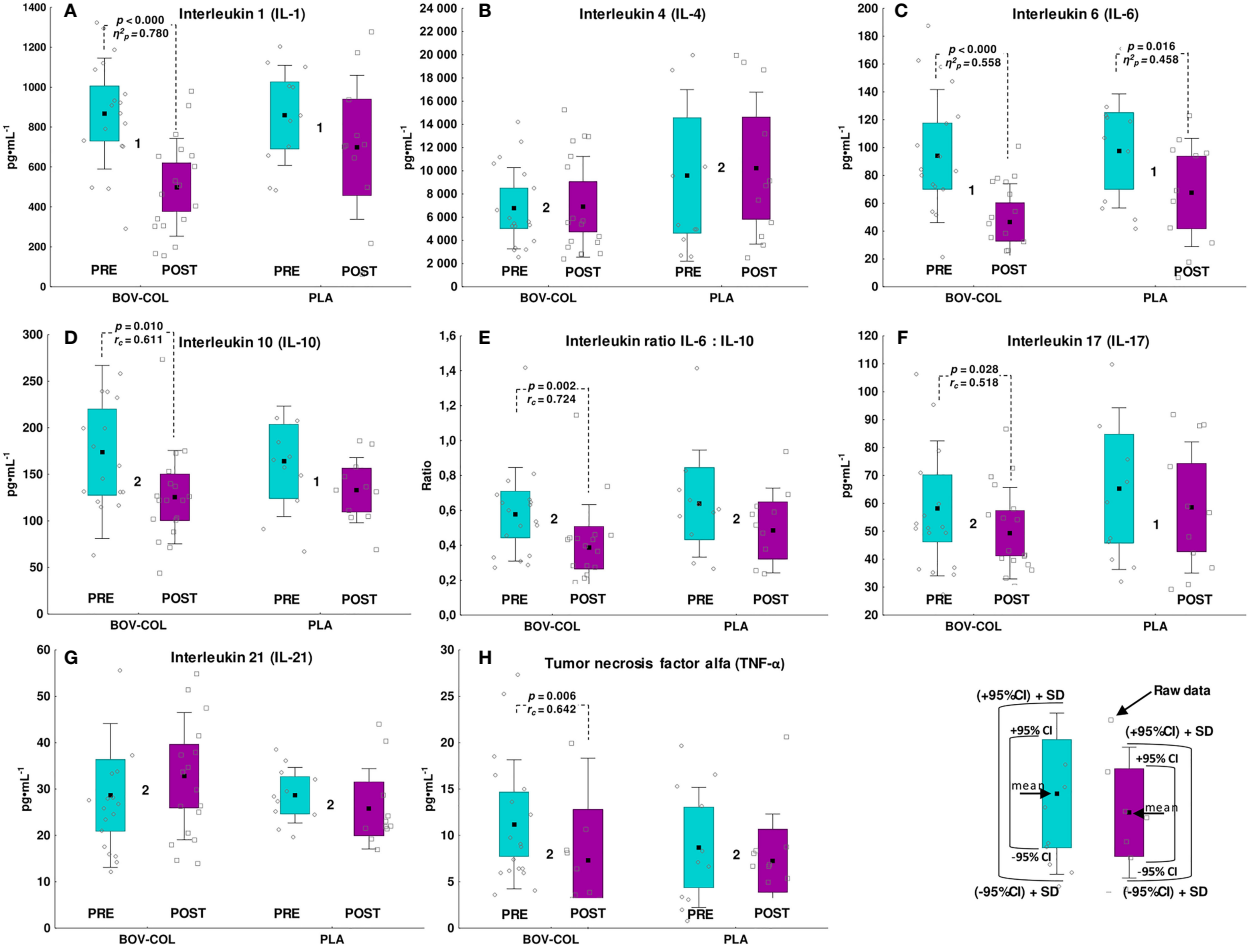
Blood concentration of inflammatory markers: **(A)** interleukin 1, **(B)** interleukin 4, **(C)** interleukin 6, **(D)** interleukin 10, **(E)** the interleukin 6 to interleukin 10 ratio, **(F)** interleukin 17, **(G)** interleukin 21, and **(H)** tumor necrosis factor alpha. The data are expressed as the mean (square), 95% confidence interval (box), 95% CI + one standard deviation (whisker), and raw data. ^1^The data were analyzed with one-way analysis of variance with repeated measurements. ^2^The data were analyzed with the Wilcoxon signed-rank test. Between-group differences (BOV-COL vs. PLA) were analyzed with the *T*-test for independent variables (there were no differences between the groups at any time point).

There were no PRE or POST supplementation differences in the inflammatory marker concentrations between the BOV-COL and PLA groups (**Figures 3A–G**). However, after BOV-COL treatment, the concentrations of IL-1 (*p* < 0.000; *η* ^2^ *_p_* = 0.780; **Figure 3A**), IL-6 (*p* < 0.000; *η* ^2^ *_p_* = 0.558; **Figure 3C**), IL-10 (*p* = 0.010; *r_c_* = 0.611; **Figure 3D**), the IL-6:IL-10 ratio (*p* = 0.002; *r_c_* = 0.724; **Figure 3E**), IL-17 (*p* = 0.028; *r_c_* = 0.518; **Figure 3F**), and TNF-α (*p* = 0.006; *r_c_* = 0.642; **Figure 3H**) were significantly decreased compared with the PRE evaluation. In the PLA group, only the IL-6 concentration was lower (*p* = 0.016; *η* ^2^ *_p_* = 0.458) at the POST evaluation compared with the PRE evaluation (**Figure 3C**). However, there were no differences in the absolute changes of inflammatory markers between the BOV-COL and PLA groups (**Supplementary Table 2**).

### 3.3 Neurotransmitters

There were no differences in the PRE or POST supplementation SER and DOP concentrations between the BOV-COL and PLA groups (**Table 2**). There were no PRE vs. POST differences in the SER or DOP concentrations in either group. Finally, there were no differences in the absolute changes in SER or DOP between the BOV-COL and PLA groups (**Supplementary Table 2**).

**Table 2:** Blood concentrations of neurotransmitters.

Indicator	Unit	BOV-COL_PRE_	BOV-COL_POST_	*p*_PRE vs. POST_ *p*-value *η* ^2^_p_ or *r_c_*	PLA_PRE_	PLA_POST_	*p*_PRE vs. POST_ *p*-value *η* ^2^_p_ or *r_c_*	*p_PRE_* BOV-COL vs. PLA	*p_POST_* BOV-COL vs. PLA
SER	ng·mL^-1^	19.5 ± 4.8	20.0 ± 5.0	0.711^2^	17.8 ± 2.9	18.5 ± 3.8	0.257^1^	0.574^4^	0.398^3^
		(17.1–21.9)	(17.5–22.5)	0.087	(15.8–19.8)	(15.9–21.0)	0.127	0.131	0.329
DOP	ng·mL^-1^	2.9 ± 1.2	2.8 ± 1.5	0.286^2^	2.3 ± 0.7	2.1 ± 0.6	0.230^1^	0.100^3^	0.144^4^
		(2.3–3.5)	(2.1–3.5)	0.251	(1.8–2.7)	(1.7–2.6)	0.141	0.651	0.333

The results are expressed as the mean ± standard deviation and 95% confidence interval (in parentheses). Abbreviations: DOP, dopamine; SER, serotonin. ^1^The data were analyzed with one-way analysis of variance with repeated measurements; the effect size is expressed as partial eta-squared (*η^2^_p_*). ^2^The data were analyzed with the Wilcoxon signed-rank test; the effect size is expressed as the rank correlation coefficient (*r_c_*). ^3^The data were analyzed with the *T*-test for independent variables; the effect size is expressed as Cohen’s *d*. ^4^The data were analyzed with the Mann–Whitney *U*-test; the effect size is expressed as Glass’s rank-biserial correlation coefficient (*r_g_*).

### 3.4 Correlations between neurotransmitters and inflammatory markers

At the PRE evaluation the in BOV-COL group, there were positive correlations between: *(a)* SER and DOP (*r* = 0.552; *p* = 0.018; **Table 3**); *(b)* TNF-α and IL-17 (*r* = 0.725; *p* = 0.001); *(c)* IL-1 and IL-6 (*r* = 0.745; *p* < 0.000), IL-10 (*r* = 0.649; *p* = 0.004), IL-17 (*r* = 0.529; *p* = 0.024), and IL-21 (*r* = 0.647; *p* = 0.004); *(d)* IL-4 and IL-10 (*r* = 0.476; *p* = 0.046); *(e)* IL-6 and IL-17 (*r* = 0.474; *p* = 0.047) and the IL-6:IL-10 ratio (*r* = 0.606; *p* = 0.008); and *(f)* IL-21 and IL-10 (*r* = 0.771; *p* < 0.000) and IL-17 (*r* = 0.715; *p* = 0.001). In turn, in the PLA group at the PRE evaluation, there were negative relationships between: *(a)* DOP and IL-6 (*r* = -0.626; *p* = 0.040; **Table 3**), IL-10 (*r* = -0.686; *p* = 0.020), and IL-21 (*r* = -0.653; *p* = 0.029); and positive relationships between: *(a)* TNF-α and IL-6 (*r* = 0.717; *p* = 0.013) and the IL-6:IL-10 ratio (*r* = 0.718; *p* = 0.013); *(b)* IL-1 and IL-6 (*r* = 0.881; *p* < 0.000); *(c)* IL-10 and IL-17 (*r* = 0.631; *p* = 0.038); and *(d)* IL-17 and IL-21 (*r* = 0.626; *p* = 0.039).

**Table 3:** Correlations between neurotransmitters and inflammatory markers at the PRE and POST evaluations.

Blood markers		BOV-COL		PLA	
		PRE	POST	PRE	POST
**SER vs. DOP**	***r*** ***p***	**0.552^1^** **0.018**	**0.503^1^** **0.034**	- -	- -
**DOP vs. IL-6**	***r*** ***p***	- -	- -	**-0.626^2^** **0.040**	- -
**DOP vs. IL-10**	***r*** ***p***	- -	- -	**-0.686^2^** **0.020**	- -
**DOP vs. IL-21**	***r*** ***p***	- -	- -	**-0.653^2^** **0.029**	- -
**TNF-α vs. IL-6**	***r*** ***p***	- -	- -	**0.717^2^** **0.013**	- -
**TNF-α vs. IL-6:IL-10**	***r*** ***p***	- -	- -	**0.718^1^** **0.013**	- -
**TNF-α vs. IL-17**	***r*** ***p***	**0.725^1^** **0.001**	- -	- -	- -
**IL-1 vs. IL-6**	***r*** ***p***	**0.745^2^** **0.000**	**0.810^2^** **0.000**	**0.881^2^** **0.000**	**0.873^2^** **0.000**
**IL-1 vs. IL-10**	***r*** ***p***	**0.649^1^** **0.004**	- -	- -	**0.671^2^** **0.024**
**IL-1 vs. IL-6:IL-10**	***r*** ***p***	- -	**0.536^1^** **0.022**	- -	**0.609^1^** **0.047**
**IL-1 vs. IL-17**	***r*** ***p***	**0.529^1^** **0.024**	- -	- -	- -
**IL-1 vs. IL-21**	***r*** ***p***	**0.647^1^** **0.004**	- -	- -	- -
**IL-4 vs. IL-10**	***r*** ***p***	**0.476^1^** **0.046**	- –	- -	- -
**IL-6 vs. IL-6:IL-10**	***r*** ***p***	**0.606^1^** **0.008**	**0.804^1^** **0.000**	- -	**0.845^1^** **0.001**
**IL-6 vs. IL-10**	***r*** ***p***	**-** **-**	- -	- -	**0.717^2^** **0.013**
**IL-6 vs. IL-17**	***r*** ***p***	**0.474^1^** **0.047**	- -	- -	- -
**IL-10 vs. IL-17**	***r*** ***p***	**-** **-**	**-** **-**	**0.631^2^** **0.038**	- -
**IL-10 vs. IL-21**	***r*** ***p***	**0.771^1^** **0.000**	**-** **-**	- -	- -
**Il-17 vs. IL-21**	***r*** ***p***	**0.715^1^** **0.001**	- -	**0.626^2^** **0.039**	- -

DOP, dopamine; IL-1, interleukin 1; IL-4, interleukin 4; IL-6, interleukin 6; IL-10, interleukin 10; IL-17, interleukin 17; IL-21, interleukin 21; SER, serotonin; TNF-α, tumor necrosis factor alpha. ^1^The data were analyzed with Spearman’s rank correlation. ^2^The data were analyzed with Pearson correlation and linear regression analysis. Bold values refer to statistically significant differences/correlations.

At the POST evaluation, the absolute number of correlations was lower, especially in the BOV-COL group. In the BOV-COL group, there were positive correlations between: *(a)* DOP and SER (*r* = 0.503; *p* = 0.034; **Table 3**); *(b)* IL-1 and IL-6 (*r* = 0.810; *p* < 0.000) and the IL-6:IL-10 ratio (*r* = 0.536; *p* = 0.022); and *(c)* IL-6 and the IL-6:IL-10 ratio (*r* = 0.804; *p* < 0.000). In PLA group, IL-1 was positively associated with *(a)* IL-6 (*r* = 0.873; *p* < 0.000), IL-10 (*r* = 0.671, *p* = 0.024), and the IL-6:IL-10 ratio (*r* = 0.609; *p* = 0.047), while IL-6 was positively associated with *(b)* IL-10 (*r* = 0.717; *p* = 0.013) and the IL-6:IL-10 ratio (*r* = 0.845; *p* = 0.001).

### 3.5 Correlations between the depression scales and inflammatory markers and neurotransmitters

Before the supplementation, there was a positive relationship between the HDRS-17 score and the IL-4 concentration in the PLA group. Apart from that, there were no other significant relationships between the BDI-II or HDRS-17 score and the inflammatory markers, SER, or DOP in the BOV-COL or PLA groups at any time point (**Table 4**).

**Table 4:** Correlations between the BDI-II and HDRS-17 scores and neurotransmitters and inflammatory markers at the PRE and POST evaluations.

			SER ng·mL^-1^	DOP ng·mL^-1^	TNF-α pg·mL^-1^	IL-1 pg·mL^-1^	IL-4 pg·mL^-1^	IL-6 pg·mL^-1^	IL-10 pg·mL^-1^	IL-6:IL-10	IL-17 pg·mL^-1^	IL-21 pg·mL^-1^
PRE												
**HDRS-17**	**BOV-COL**	***r*** ***p***	0.082^1^ 0.747	0.098^2^ 0.698	0.152^1^ 0.546	0.303^2^ 0.222	0.117^2^ 0.645	0.314^2^ 0.204	0.201^1^ 0.424	0.207^1^ 0.409	0.175^1^ 0.487	0.218^1^ 0.386
	**PLA**	***r*** ***p***	0.025^2^ 0.943	0.066^2^ 0.847	-0.006^2^ 0.987	-0.128^2^ 0.708	**0.836^1^** **0.001**	-0.042^2^ 0.902	-0.130^2^ 0.703	0.110^1^ 0.748	0.131^2^ 0.702	0.380^2^ 0.249
**BDI-II**	**BOV-COL**	***r*** ***p***	0.055^1^ 0.829	-0.120^2^ 0.637	-0.196^1^ 0.437	0.064^2^ 0.802	0.141^2^ 0.576	0.102^2^ 0.687	-0.173^1^ 0.493	0.356^1^ 0.147	-0.141^1^ 0.578	-0.118^1^ 0.641
	**PLA**	***r*** ***p***	0.009^2^ 0.980	0.208^2^ 0.539	-0.234^2^ 0.488	-0.506^2^ 0.113	0.292^1^ 0.383	-0.363^2^ 0.272	0.022^2^ 0.950	-0.311^1^ 0.353	-0.137^2^ 0.689	-0.026^2^ 0.940
POST												
**HDRS-17**	**BOV-COL**	***r*** ***p***	0.141^2^ 0.576	-0.022^1^ 0.931	0.042^1^ 0.870	-0.034^2^ 0.893	0.011^1^ 0.964	0.152^2^ 0.546	0.160^2^ 0.527	-0.001^1^ 0.997	-0.143^2^ 0.572	-0.011^2^ 0.964
	**PLA**	***r*** ***p***	-0.116^2^ 0.733	-0.009^2^ 0.978	-0.371^1^ 0.262	-0.325^2^ 0.329	0.035^2^ 0.92	-0.098^2^ 0.774	0.190^2^ 0.575	-0.256^1^ 0.447	-0.373^2^ 0.259	-0.124^1^ 0.259
**BDI-II**	**BOV-COL**	***r*** ***p***	0.169^2^ 0.503	0.065^1^ 0.797	0.080^1^ 0.754	0.066^2^ 0.794	0.057^1^ 0.823	0.234^2^ 0.351	-0.115^2^ 0.651	0.303^1^ 0.222	-0.326^2^ 0.186	0.334^2^ 0.176
	**PLA**	***r*** ***p***	-0.330^2^ 0.322	0.286^2^ 0.394	-0.349^1^ 0.293	-0.541^2^ 0.086	-0.073^2^ 0.831	-0.320^2^ 0.337	-0.145^2^ 0.671	-0.385^1^ 0.242	-0.397^2^ 0.226	0.055^1^ 0.872

BDI-II, Beck Depression Inventory; DOP, dopamine; HDRS-17, 17-item Hamilton Scale for Depression Rating; IL-1, interleukin 1; IL-4, interleukin 4; IL-6, interleukin 6; IL-10, interleukin 10; IL-17, interleukin 17, IL-21, interleukin 21; SER, serotonin; TNF-α, tumor necrosis factor alpha. ^1^The data were analyzed with Spearman’s rank correlation. ^2^The data were analyzed with Pearson correlation and linear regression analysis. Bold values refer to statistically significant differences/correlations.

### 3.6 Blood hematological evaluation

Apart from PLCR, which was significantly higher in the PLA group (*p* = 0.032; *d* = -0.863), there were no other differences in hematological blood indices between the studied groups at the PRE evaluation (**Table 5**). However, at the POST evaluation, WBC (*p* = 0.033; *r_g_* = -0.485) and MON (*p* = 0.037; *r_g_* = -0.475) counts were higher in the PLA group compared with the BOV-COL group. In the BOV-COL group, the WBC count (*p* = 0.011; *r_c_* = 0.600), LYM count (*p* = 0.029; *η* ^2^ *_p_* = 0.252), and GRA (count: *p* = 0.012; *r_c_* = 0.590; percentage: *p* = 0.017; *η* ^2^ *_p_* = 0.293) were significantly lower, and MON (count: *p* = 0.001; *r_c_* = 0.816; percentage: *p* < 0.000; *η* ^2^ *_p_* = 0.833) was significantly higher at the POST evaluation compared with the PRE evaluation. In the PLA group, the LYM count (*p* = 0.045; *η* ^2^ *_p_* = 0.345) and MPV (*p* = 0.024; *η* ^2^ *_p_* = 0.415) were lower, and MON (count: *p* < 0.000; *η* ^2^ *_p_* = 0.847 and percentage: *p* < 0.000; *r_c_* = 0.878) was higher at the POST evaluation compared with the PRE evaluation. In both groups, the HGB concentration (BOV-COL: *p* < 0.000; *η* ^2^ *_p_* = 0.808; PLA: *p* < 0.000; *η* ^2^ *_p_* = 0.929), MCH (BOV-COL: *p* < 0.000; *r_c_* = 0.879; PLA: *p* < 0.000; *r_c_* = 0.884), and MCHC (BOV-COL: *p* < 0.000; *η* ^2^ *_p_* = 0.807; PLA: *p* < 0.000; *η* ^2^ *_p_* = 0.934) were lower, and the RDW-S (BOV-COL: *p* = 0.008; *r_c_* = 0.626; PLA: *p* = 0.017; *η* ^2^ *_p_* = 0.447), PLT (BOV-COL: *p* < 0.000; *η* ^2^ *_p_* = 0.674; PLA: *p* = 0.002; *η* ^2^ *_p_* = 0.641), and PCT (BOV-COL: *p* < 0.000; *r_c_* = 0.878; PLA: *p* = 0.002; *η* ^2^ *_p_* = 0.621) were higher at the POST evaluation compared with the PRE evaluation. Apart from MPV (*p* = 0.030; *d* = 0.877) which was significantly more reduced in the PLA compared to the BOV-COL group, there were no differences in absolute changes in the blood hematological indices between the BOV-COL and PLA groups (**Supplementary Table 3**).

**Table 5:** Blood hematological indices.

Indicator	Units	BOV-COL_PRE_	BOV-COL_POST_	*p*_PRE vs. POST_ *p*-value *η* ^2^_p_ or *r_c_*	PLA_PRE_	PLA_POST_	*p*_PRE vs. POST_ *p*-value *η* ^2^_p_ or *r_c_*	PRE BOV-COL vs. PLA *p*-value Cohen’s *d* or *r_g_*	POST BOV-COL vs. PLA *p*-value Cohen’s *d* or *r_g_*
WBC	10^9^·L^-1^	8.4 ± 1.7 (7.6–9.2)	7.6 ± 2.5 (6.3–8.8)	**0.011^2^** **0.600**	9.3 ± 2.2 (7.8–10.8)	9.1 ± 2.5 (7.5–10.8)	0.807^1^ 0.006	0.226^3^ -0.474	**0.033^4^** **-0.485**
LYM	10^9^·L^-1^	2.8 ± 0.8 (2.4–3.2)	2.5 ± 0.6 (2.2–2.8)	**0.029^1^** **0.252**	3.2 ± 0.8 (2.6–3.7)	2.8 ± 0.6 (2.4–3.2)	**0.045^1^** **0.345**	0.237^3^ -0.463	0.236^3^ -0.464
MON	10^9^·L^-1^	0.4 ± 0.1 (0.4–0.5)	0.6 ± 0.2 (0.5–0.7)	**0.001^2^** **0.816**	0.5 ± 0.1 (0.4–0.6)	0.7 ± 0.2 (0.6 ± 0.8)	**0.000^1^** **0.847**	0.225^3^ -0.475	**0.037^4^** **-0.475**
GRA	10^9^·L^-1^	5.2 ± 1.4 (4.5–5.9)	4.4 ± 2.2 (3.4–5.5)	**0.012^2^** **0.590**	5.7 ± 1.9 (4.4–6.9)	5.6 ± 2.4 (4.0–7.3)	0.424^2^ 0.241	0.702^4^ -0.091	0.092^4^ -0.384
LYM	%	33.4 ± 7.6 (29.6–37.2)	35.1 ± 8.7 (30.8–39.4)	0.291^1^ 0.065	34.5 ± 8.2 (29.0–40.0)	32.6 ± 10.5 (25.5–39.6)	0.442^1^ 0.060	0.715^3^ -0.141	0.491^3^ 0.267
MON	%	5.1 ± 0.9 (4.6–5.6)	8.1 ± 1.5 (7.3–8.8)	**0.000^1^** **0.833**	5.0 ± 1.2 (4.2–5.8)	8.0 ± 1.1 (7.3–8.8)	**0.000^1^** **0.878**	0.925^3^ 0.036	0.952^3^ 0.023
GRA	%	61.5 ± 7.8 (57.6–65.4)	56.9 ± 9.0 (52.4–61.4)	**0.017^1^** **0.293**	60.5 ± 7.9 (55.1–65.8)	59.4 ± 10.7 (52.2–66.6)	0.694^1^ 0.016	0.726^3^ 0.136	0.497^3^ -0.263
RBC	10^12^·L^-1^	5.6 ± 0.2 (5.5–5.8)	5.6 ± 0.3 (5.5–5.7)	0.557^2^ 0.139	5.6 ± 0.2 (5.5–5.8)	5.6 ± 0.2 (5.5–5.7)	0.495^1^ 0.048	0.805^4^ 0.061	0.946^4^ 0.020
HCT	%	0.428 ± 0.027 (0.414–0.441)	0.427 ± 0.032 (0.411–0.443)	0.945^1^ 0.000	0.434 ± 0.030 (0.413–0.454)	0.431 ± 0.025 (0.414–0.448)	0.266^2^ 0.335	0.4184 -0.187	0.747^3^ -0.125
HGB	mmol·L^-1^	10.7 ± 0.2 (10.6–10.8)	10.2 ± 0.2 (10.1–10.3)	**0.000^1^** **0.808**	10.7 ± 0.2 (10.6–10.8)	10.1 ± 0.1 (10.0–10.2)	**0.000^1^** **0.929**	0.334^3^ 0.376	0.082^3^ 0.691
MCV	fL	89.0 ± 3.7 (87.1–90.8)	89.2 ± 3.9 (87.3–91.2)	0.717^2^ 0.085	89.0 ± 3.3 (86.8–91.2)	89.3 ± 3.1 (87.2–91.4)	0.475^1^ 0.052	0.787^4^ 0.066	0.746^4^ -0.020
MCH	fmol	1.9 ± 0.1 (1.9–2.0)	1.8 ± 0.1 (1.8–1.9)	**0.000^2^** **0.879**	1.9 ± 0.1 (1.8–2.0)	1.8 ± 0.1 (1.8–1.8)	**0.000^1^** **0.884**	0.500^4^ 0.157	0.406^4^ 0.192
MCHC	mmol·L^-1^	21.5 ± 0.3 (21.3–21.6)	20.4 ± 0.4 (20.2–20.6)	**0.000^1^** **0.807**	21.3 ± 0.3 (21.1–21.5)	20.2 ± 0.3 (20.0–20.3)	**0.000^1^** **0.934**	0.309^3^ 0.397	0.068^3^ 0.726
RDW-C	%	11.6 ± 1.0 (11.2–12.1)	11.8 ± 1.2 (11.3–12.4)	0.446^2^ 0.180	11.9 ± 0.3 (11.7–12.1)	12.1 ± 0.6 (11.7–12.6)	0.287^1^ 0.112	0.430^3^ -0.306	0.150^4^ -0.328
RDW-S	fL	43.8 ± 2.2 (42.7–44.9)	46.2 ± 3.3 (44.5–47.8)	**0.008^2^** **0.626**	44.7 ± 2.3 (43.1–46.2)	46.7 ± 2.9 (44.7–48.7)	**0.017^1^** **0.447**	0.340^3^ -0.371	0.301^4^ 0.237
PLT	10^9^·L^-1^	236.9 ± 58.6 (207.8–266.0)	287 ± 81.9 (246.3–327.7)	**0.000^1^** **0.674**	211.9 ± 55.2 (174.8–249.0)	271.2 ± 94.4 (207.8–334.6)	**0.002^1^** **0.641**	0.264^3^ 0.436	0.639^3^ 0.182
MPV	fL	9.0 ± 0.5 (8.7–9.2)	8.9 ± 0.5 (8.6–9.2)	0.250^1^ 0.077	9.5 ± 0.9 (8.9–10.1)	9.1 ± 0.8 (8.6–9.7)	**0.024^1^** **0.415**	0.053^3^ -0.772	0.367^3^ -0.351
PCT	cl·L^-1^	0.21 ± 0.05 (0.19–0.23)	0.25 ± 0.06 (0.22–0.28)	**0.000^2^** **0.878**	0.20 ± 0.04 (0.17–0.22)	0.24 ± 0.07 (0.20–0.29)	**0.002^1^** **0.621**	0.515^4^ 0.152	0.706^3^ 0.146
PDW	%	14.5 ± 1.3 (13.9–15.2)	14.7 ± 1.2 (14.0–15.3)	0.803^1^ 0.004	14.7 ± 1.0 (14.0–15.4)	14.1 ± 1.1 (13.4–14.9)	0.075^2^ 0.429	0.731^3^ -0.133	0.258^4^ 0.323
PLCR	%	16.5 ± 3.5 (14.8–18.3)	16.7 ± 3.9 (14.7–18.7)	0.749^1^ 0.006	20.7 ± 6.6 (16.3–25.1)	18.8 ± 5.6 (15.0–22.6)	0.175^1^ 0.176	**0.032^3^** **-0.863**	0.244^3^ -0.456

The results are expressed as the mean ± standard deviation and 95% confidence interval (in parentheses). GRA, granulocytes; HCT, hematocrit; HGB, hemoglobin; LYM, lymphocytes; MCH, mean corpuscular hemoglobin mass; MCHC, mean corpuscular hemoglobin concentration; MCV, mean corpuscular volume; MON, monocytes; MPV, mean platelet volume; PCT, platelet hematocrit; PDW, platelet distribution width; PLT, platelet count; PLCR, platelet large cell ratio; RBC, red blood cells; RDW-C, red blood cells distribution width – coefficient of variation; RDW-S, red blood cells distribution width – standard deviation; WBC, white blood cells. ^1^The data were analyzed with one-way analysis of variance with repeated measurements; the effect size is expressed as partial eta-squared (*η^2^_p_*). ^2^The data were analyzed with the Wilcoxon signed-rank test; the effect size is expressed as the rank correlation coefficient (*r_c_*). ^3^The data were analyzed with the *T*-test for independent variables; the effect size is expressed as Cohen’s *d*. ^4^The data were analyzed with the Mann–Whitney *U*-test; the effect size is expressed as Glass’s rank-biserial correlation coefficient (*r_g_*). Bold values refer to statistically significant differences/correlations.

## 4 Discussion

This study is the first to innovatively verify the effectiveness of BOV-COL supplementation as a supportive treatment for depression in hospitalized patients with co-occurring SUD. We hypothesized that ingestion of BOV-COL triggers beneficial alternations in the pro-/anti-inflammatory status of the body, hematological blood indices, as well as selected neurotransmitters, and eventually improves the outcomes of depression treatment based on the BDI-II and HDRS-17. The mean BDI-II score was significantly reduced at the POST evaluation in both groups. However, the mean HDRS-17 score was decreased in the BOV-COL group, but not in the PLA group. The latter finding suggests the additional benefits of BOV-COL supplementation over standard depression treatment (and PLA supplementation). In the BOV-COL group, there were more alternations in cytokine concentrations (reduction in IL-1, IL-6, IL-10, the IL-6:IL-10 ratio, IL-17, and TNF-α) than in the PLA group (reduction in IL-6). Moreover, at the POST evaluation, there were more pronounced and favorable alternations in total count and differentials of WBC subsets in the BOV-COL group compared with the PLA group. Interestingly, in both groups, there were unfavorable changes in hematological markers of iron metabolism—a reduction in the hemoglobin concentration, as well as MCH and MCHC. However, the aforementioned markers were within the reference ranges at the PRE and POST evaluations in both groups. There were no changes in neurotransmitter concentrations.

It should also be underlined that there are no studies on the supplementation of BOV-COL in patients with depression and ratings of depression. However, other nutrient and dietary supplements have been thoroughly examined as part of adjunctive depression therapy. According to the recent review by Thurfah et al. (19), PUFA (mainly a combination of docosahexaenoic and eicosapentaenoic acids), vitamin D, and probiotics are the most common and promising supplements implemented in clinical studies to reduce depressive symptoms as measured by the BDI-II and HDRS-17.

The current study indicated a significant decrease in most the of evaluated cytokines in the BOV-COL group (IL-1, IL-6, IL-10, the IL-6:IL-10 ratio, IL-17, and TNF-α), and a reduction in only IL-6 in the PLA group. IL-6 is a pleiotropic cytokine that exhibits both pro- and anti-inflammatory actions (44). However, IL-6 is the most consistently elevated cytokine in the blood of patients with MDD (45), and the IL-6 concentration may serve as a predictive biomarker in depression treatment outcomes (plasma IL-6 is observed to be higher in antidepressant-resistant patients compared with antidepressant-responsive patients) (46). Simultaneously, in antidepressant non-responders, peripheral levels of IL-6 positively correlate with symptoms severity (47). Pharmacotherapy of depression may have a diverse impact on cytokines levels. Overall, antidepressants cause a decrease in peripheral concentrations of IL-6, IL-10, TNF-α, and CCL-2 (13, 45). A decrease in a broad range of inflammatory markers during antidepressant treatment may correlate with specific dimensions of depression symptoms (7). Liu et al. (48) found that responders to antidepressants had lower baseline IL-8 levels and exhibited a greater decrease in TNF-α compared with non-responders. On the other hand, antipsychotic drugs, especially those with the highest risk of body mass gain (i.e., olanzapine), cause substantial increases in blood levels of pro-inflammatory cytokines; similarly, mood stabilizers including lithium and carbamazepine have been linked with an increase in peripheral levels of cytokines (45). In the current study, those particular groups of drugs were prescribed to three of the study participants.

There have been some other studies on BOV-COL supplementation and inflammatory markers in various population groups. Skarpańska-Stejnborn et al. (49) evaluated young female basketball players and reported a significant increase in the plasma IL-10 concentration 3 hours after a maximum exercise test on a treadmill after 6 months of supplementation in the PLA group, but not in the BOV-COL group (8 *x* 400 mg = 3200 mg of BOV-COL per day). Simultaneously, other immune markers such as IL-1α, IL-2, IL-13, and TNF-α did not change. In an *ex vivo* study using immune cells from colorectal cancer patients, Gasser et al. (50) examined the effects of colostrum preparation to determine the effect of colostrum polyvalent Ig (KMP01D) on the inflammatory activity of patient-derived immune cells. The authors observed increased expression of IL-10 and IL-13 and decreased expression of IL-1β, IL-6, interferon gamma (IFN-γ), TNF-α, IL-12, and IGF-1 in peripheral blood mononuclear cells (PBMCs) derived from patients with colorectal cancer. A pilot study of 5-week supplementation with a BOV-COL product (CBP; 0.15 g_CBP_·lb^-1^·day^-1^) alone or in combination with a probiotic (*Bifidobacterium infantis*; CBP *+ B. infantis*) in children with autism and comorbid gastrointestinal symptoms reported a reduction in the frequency of CD4^+^ producing IL-13 after CBP + *B. infantis* co-supplementation and a reduction in the frequency of CD8^+^ producing TNF-α after CBP supplementation in PBMCs (51). Simultaneously, there were no significant changes in the frequency of CD4^+^ or CD8^+^ T cells expressing IFN-γ, IL-17, or IL-6. Kotsis et al. (52) demonstrated that a 6-week BOV-COL supplementation (8 *x* 400 mg = 3200 mg of BOV-COL per day) attenuated the post-exercise increase in IL-6, in contrast to whey protein supplementation. Cieślicka et al. (53) evaluated female athletes and found that the 6-month supplementation of BOV-COL resulted in a lower resting IL-6 concentration compared with the control group, yet BOV-COL supplementation (8 *x* 400 mg = 3200 mg of BOV-COL per day) was related to a significant post-exercise increase in IL-6, while PLA was not. Shing et al. (54) studied the effects of 5-week BOV-COL supplementation (10 g·day^-1^; 20% IgG) on immune variables in highly trained cyclists. They revealed similar between-group results regarding the IFN-γ, TNF-α, IL-6, IL-10, and IL-12p40 concentrations. Similarly, Carol et al. (55) observed no differences between the effect of a 10-day BOV-COL supplementation (freeze-dried BOV-COL with IgG ~65 g·L^-1^; 2 *x* 12.5 g·day^-1^) or skim-milk powder supplementation on any of the investigated variables, including IL-6, IL-10, IL-1 receptor agonist, CRP, IFN-γ, IL-1α, IL-8, and TNF-α. It should be underlined that the pro-/anti-inflammatory disturbances that occurred in the patients with depression and SUD that we evaluated, compared with the patients studied in the previous investigations, were evoked by different triggers, and the degree of their severity varied between the groups. Thus, the results of BOV-COL treatment regarding the pro-/anti-inflammatory status may be still conflicting. Moreover, the effect of BOV-COL supplementation on the pro-/anti-inflammatory status of the body should be evaluated by considering the interactions and a dynamic balance between particular cytokines. For example, we found a considerably reduced absolute number of significant correlations between inflammatory markers at the POST evaluation compared with the PRE evaluation in the BOV-COL group (9 vs. 2), but not in the PLA group. This finding, together with a greater reduction in the concentration of most cytokines in the BOV-COL group, clearly and undeniably indicate more pronounced mitigation of inflammation in the BOV-COL group than the PLA group.

In our study, we found no differences in SER and DOP concentrations between the groups. There are no other studies on BOV-COL supplementation on neurotransmitters in patients with depression.

Regarding the hematological measurements, we observed significantly higher concentrations of WBC and MON at the POST evaluation in the PLA group. Although these values remained in the reference ranges, the results suggest fewer immune perturbations in the BOV-COL group. Interestingly, in both of the studied groups we observed a significant reduction in the HGB concentration, as well as a decrease in MCH and MCHC, and an increase in RDW-S. These changes may indicate an increased risk of developing anemia in the study participants. It has been reported that depression and antidepressant use (specifically selective serotonin reuptake inhibitors [SSRIs] and serotonin-norepinephrine reuptake inhibitors [SNRIs]) are independently associated with low HGB concentrations (56). Out of 29 patients who completed the supplementation protocol, 25 patients were treated with antidepressants—10 of them with SSRIs/SNRIs. Moreover, there are no studies on the effect of BOV-COL supplementation on the hematological status in patients with depression. Nevertheless, there are several studies on physically active populations. In our systematic review and meta-analysis (22), we specifically analyzed the immunological outcomes of BOV-COL supplementation in trained and physically active people. We found that BOV-COL supplementation has no or a fairly low impact on improving the concentration of LYM and neutrophils. Skarpańska-Stejnborn et al. (49) evaluated young female basketball players and also found no effect of BOV-COL on WBC, LYM, MON, and GRA. Cieślicka et al. (53) revealed no differences in RBC, HGB, and HTC at rest between their study groups (BOV-COL vs. PLA). In their *in vivo* immunity study, Jones et al. (57) indicated no significant group or interaction effects for total or differential WBC counts. March et al. (58) also found no effect of BOV-COL supplementation on WBC, neutrophils, LYM, and MON following exercise in the heat.

In our study, there were no significant correlations between the depression scale outcomes and inflammatory markers or neurotransmitters at the POST evaluation in the BOV-COL group. Kofod et al. (7) found no baseline correlations between any of the 27 studied inflammatory markers and differential severity based on the overall Montgomery–Åsberg Depression Rating Scale, although several of the inflammatory markers correlated (small correlations) with the differential severity of specific symptom dimensions. The results support a previous finding of no correlation between CRP, IL-6, and TNF-α and overall depression severity, but rather with specific symptoms (7, 59). Nevertheless, there are different results from studies concerning correlations between a reduction in inflammatory cytokines and an improvement in depressive symptoms or an improvement in depression scores independent of changes in several inflammatory cytokines or an increase in inflammatory markers despite clinical improvement of the depression (7).

It should be noted that our research has some limitations. First, there was unequal participation of women and men in the study. Men have historically abused alcohol and other substances more frequently than women; however, the gender gap in SUD is narrowing (60). Moreover, it is well known that women are still less likely to enter substance use treatment (because of economic barriers, family responsibilities, etc.) compared with men relative to the prevalence of SUD in the general population (60, 61). Thus, the inequality in gender participation in the current study is not surprising. Second, there was a relatively high dropout rate. Eighteen enrolled patients did not complete the supplementation protocol; however, it needs to be emphasized that most of the dropouts (*n* = 16) were due to a discharge from the treatment rehabilitation center at the patient’s request. Thus, these dropouts were independent of the supplementation protocols. Moreover, treatment compliance problems are highly prevalent in psychiatric patients with SUD, and according to the findings by Herbeck et al. (62), out of 342 studied patients, more than 40% experienced treatment compliance problems. Our main aim was to evaluate the impact of BOV-COL supplementation on depression treatment outcomes. Still, in the studied participants depression co-occurred with SUD; hence, it was impossible to determine the isolated effect of BOV-COL on depression only. However, the approach of investigating a single rehabilitation center ensured less diversity in the depression causes (and co-occurring disorders) compared with conducting a study in free-living patients receiving treatment for depression. We did not calculate the sample size *a priori*, and we decided to conduct the investigation as a single-center study, with patients hospitalized in the same treatment center. We chose this approach to ensure that the primary and secondary outcomes could be measured in a repeatable manner, and to ensure total compliance with the supplementation protocol. Moreover, future studies might consider the effectiveness of BOV-COL supplementation according to the type of addiction, i.e. type of psychoactive substance abused. The latter would partially explain inter-individual variation in BOV-COL in patients with SUD and depression. Finally, the protocol of the current study did not include performing BDI-II and HDRS-17 evaluation in participants who dropped out from the study protocol, neither at the moment of dropping out, nor at the time-point of the anticipated completion of study protocol. Thus, we performed ITT analyses using linear interpolation for estimating the missing post-supplementation results. The ITT analyses revealed significant reduction in BDI-II and HDRS-17 after supplementation in both groups. Still, future studies must consider evaluation of end-point outcomes in participants who were lost during the treatment protocols, so that ITT analysis would provide more reliable results.

There are some undeniable strengths of our study. Thanks to conducting the investigation in one wave in all participants, we avoided the possible seasonality of the occurrence and severity of depression, which could have also influenced other outcomes. In addition, we conducted the study by including patients hospitalized in a treatment rehabilitation center. Thus, the compliance to the study protocol (the ingestion of supplemented preparations) was carefully monitored and total compliance was achieved. All patients were treated in the same center by the same medical staff and using similar treatment approaches. Thus, markedly different treatment strategies did not interfere with the effects of the implemented supplementation and did not mask potential treatment outcomes. Moreover, the single-center approach assured a standardized and repeatable manner of evaluating the severity of depression.

To summarize, there were no baseline relationships between the severity of depression—based on the BDI-II and HDRS-17 scores—and blood inflammatory markers or neurotransmitters in patients with depression and co-occurring SUD. However, a 3-month supplementation of BOV-COL as an add-on therapy substantially reduced the severity of low-grade inflammation and, compared with PLA, resulted in considerable improvement in depression treatment outcomes as assessed by the reduction in the mean HDRS-17 score. The latter association indicates that low-grade inflammation is a possible causative factor for depression. Moreover, BOV-COL supplementation mitigated the disturbances in counts and differentials of subsets of blood cells. Surprisingly, the results of the study indicate possible disturbances in iron metabolism in patients with depression and co-occurring SUD. Further investigation of nutritional add-on therapies should focus on the anti-inflammatory potential of food constituents and dietary supplements and consider improving the iron status of the body. To conclude, BOV-COL supplementation may be recommended as an effective and safe adjunct therapy in patients with depression and SUD.

## Data availability statement

The original contributions presented in the study are included in the article/**Supplementary Material**. Further inquiries can be directed to the corresponding author.

## Ethics statement

The studies involving humans were approved by Regional Medical Chamber in Gdansk (reference number KB-39/18, issued on December 20, 2018). The studies were conducted in accordance with the local legislation and institutional requirements. The participants provided their written informed consent to participate in this study.

## Author contributions

KDM: Writing – review & editing, Writing – original draft, Visualization, Validation, Supervision, Methodology, Investigation, Formal analysis, Data curation, Conceptualization. NG: Writing – review & editing, Writing – original draft, Data curation. TP: Writing – review & editing, Investigation. WO: Writing – review & editing, Investigation, Data curation. MK: Writing – review & editing, Resources, Project administration. RB: Writing – review & editing, Investigation, Conceptualization. SB: Writing – review & editing, Project administration, Funding acquisition, Conceptualization. PMN: Writing – review & editing, Writing – original draft, Visualization, Software, Investigation, Formal analysis, Data curation.
