# Supplementary material for: Bovine colostrum supplementation as a new perspective in depression and substance use disorder treatment: a randomized placebo-controlled study
Source: Front Psychiatry. 2024 Jun 18;15:1366942. doi: 10.3389/fpsyt.2024.1366942 (PMC11217880; doi:10.3389/fpsyt.2024.1366942)
Supplement: Supplementary file 1 [file DataSheet_1.pdf]

Supplementary Materials

Bovine colostrum supplementation as a new perspective in depression and substance use disorder treatment: a randomized placebo-controlled study

Krzysztof Durkalec-Michalski*, Natalia Główka, Tomasz Podgórski, Weronika Odrobny, Marcin Krawczyński, Ryszard Botwina, Stanisław Bodzicz , Paulina M. Nowaczyk

*** Correspondence:** Krzysztof Durkalec-Michalski; durkalec-michalski@awf.poznan.pl

Supplementary table 1. Baseline characteristics of all participants randomized to study protocol according to full completion (completed) of the study protocol or dropping-out (lost) from the protocol (regardless of randomization to the BOV-COL or PLA groups)

		**Unit**	**Completed**	**Lost**	***p***
		(*n*)	29	18	-
Women/men	-	(*n*)	5/24	3/15	-
MMPI-2	-	(points)	72 ± 8	68 ± 5	0.037[1]
MMPI-2 classification	Elevated	(*n*/%)	5/17	4/22	0.175[2]
	High	(*n*/%)	16/55	13/72	χ^2 = 3.482
	Very high	(*n*/%)	8/28	1/6	
Duration of the stay at the treatment center at the start of the supplementation	-	(months)	5 ± 3	6 ± 4	0.8761
BDI-II					
	-	(points)	23 ± 9	24 ± 9	0.597[1]
	No depression	(*n*/%)	6/20.5	3/17	
	Mild depression	(*n*/%)	6/20.5	2/11	0.467[2]
	Moderate depression	(*n*/%)	8/28	9/50	χ^2 = 2.547
	Severe depression	(*n*/%)	9/31	4/22	
HDRS-17					
	-	(points)	13 ± 5	14 ± 5	0.647[1]
	No depression	(*n*/%)	2/7	1/5.5	
	Mild depression	(*n*/%)	13/45	9/50	0.855[2]
	Moderate depression	(*n*/%)	11/38	5/28	χ^2 = 0.779
	Severe depression	(*n*/%)	3/10	3/16.5	

Abbreviations: BDI-II, Beck Depression Inventory; HDRS-17, 17-item Hamilton Scale for Depression Rating; MMPI-2, Minnesota Multiphasic Personality Inventory-2. [1]Data analyzed with the *T*-test for independent variables. [2]Data were analyzed with the chi-square test of independence (and results expressed as Pearson χ^2).

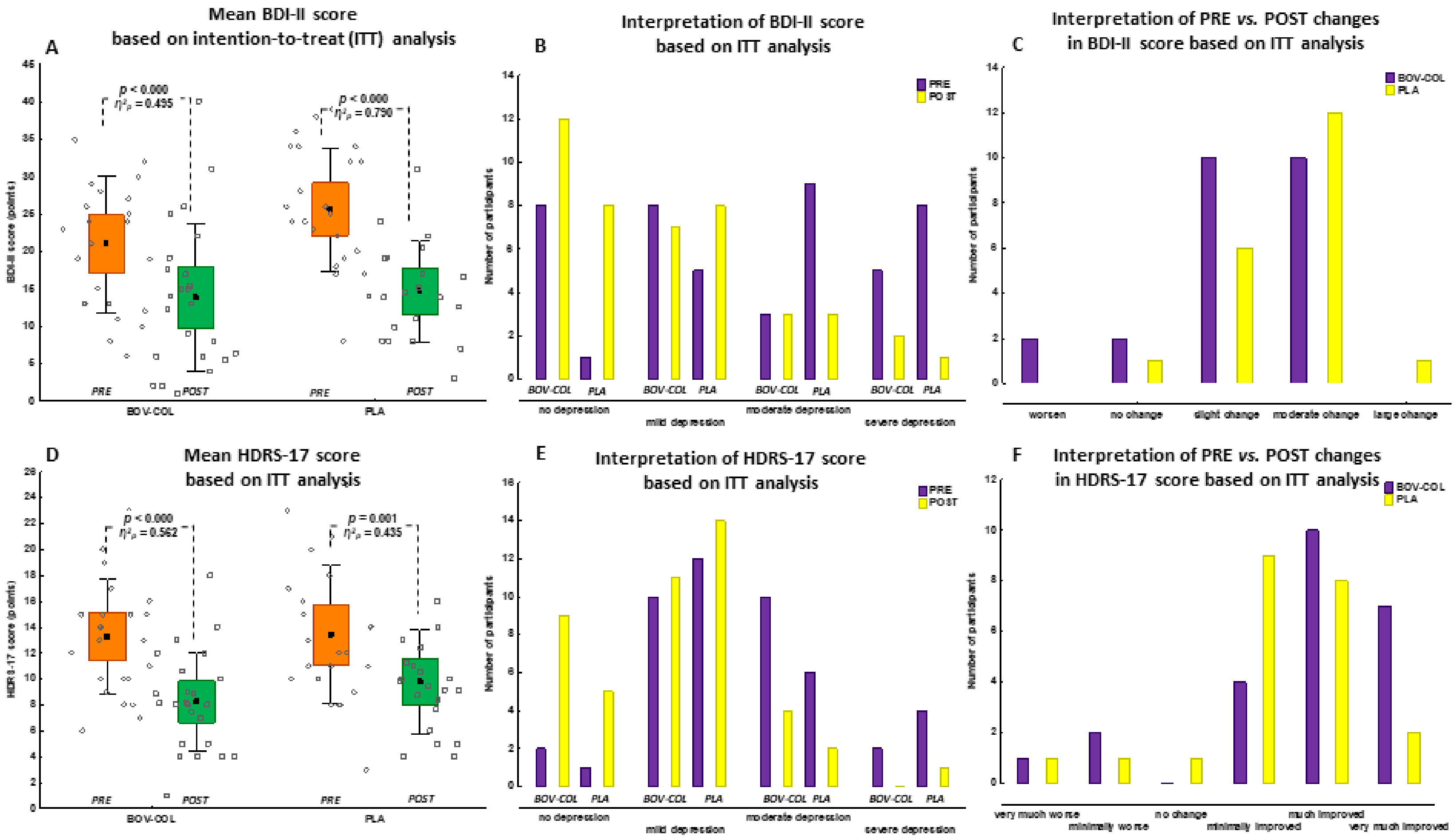

Supplementary figure 1. Results of the intention-to-treat (ITT) analysis – the mean **(a)** Beck Depression Inventory-II (BDI-II) and **(d)** 17-point Hamilton Depression Rating Scale (HDRS-17) scores; classification of depression severity according to the **(b)** BDI-II and **(e)** HDRS-17; and interpretation of PRE *vs.* POST changes in the **(c)** BDI-II and **(f)** HDRS-17. For **(a)** and **(d)**, the data are expressed as the mean (square), 95% confidence interval (box), 95% CI + one standard deviations (whisker), and raw data; the data were analyzed with one-way analysis of variance with repeated measurements (PRE *vs.* POST comparisons) or the *T*-test for independent variables (BOV-COL *vs.* PLA comparisons; no differences between the groups at any time point). For **(b)**, **(c)**, **(e)**, and **(f)**, the data are expressed as the number of participants; the data were analyzed with the chi-square test of independence and Pearson χ^2 (BOV-COL *vs.* PLA comparisons; no differences between the groups at any time point).

Supplementary table 2. Changes in neurotransmitters and inflammatory markers

Indicator	Units	BOV-COL	PLA	BOV-COL *vs.* PLA *p*-value Cohen's *d* or r_g
SER	ng·mL^{-1}	0.50 ± 3.47	0.67 ± 1.83	0.884[1]
		((-)1.23 – 2.23)	((-)0.57 – 1.90)	-0.056
DOP	ng·mL^{-1}	(-)0.11 ± 0.68	(-)0.11 ± 0.30	0.979[1]
		((-)0.45 – 0.23)	((-)0.31 – 0.09)	0.010
TNF-α	pg·mL^{-1}	(-)3.88 ± 12.67	(-)1.45 ± 4.73	0.056[2]
		((-)10.18 – 2.42)	((-)4.62 – 1.73)	-0.434
IL-1	pg·mL^{-1}	(-)370 ± 202	(-)160 ± 302	0.121[2]
		((-)470 – (-)269)	((-)363 – 43)	-0.354
IL-4	pg·mL^{-1}	126 ± 2029	627 ± 2254	0.574[2]
		((-)883 – 1135)	((-)887 – 2141)	-0.131
IL-6	pg·mL^{-1}	(-)47.4 ± 43.5	(-)29.8 ± 34.0	0.265[1]
		((-)69.0 – (-)25.7)	((-)52.7 – (-)7.0)	-0.435
IL-10	pg·mL^{-1}	(-)48.5 ± 83.5	(-)31.0 ± 54.4	0.875[2]
		((-)90 – (-)7.0)	((-)67.5 – 5.6)	0.040
IL-17	pg·mL^{-1}	(-)8.88 ± 14.82	(-)6.79 ± 20.21	0.751[1]
		((-)16.24 – (-)1.51)	((-)20.37 – 6.79)	-0.123
IL-21	pg·mL^{-1}	4.15 ± 19.64	(-)2.92 ± 12.19	0.294[1]
		((-)5.61 – 13.92)	((-)11.11 – 5.27)	0.410

Results expressed as means ± standard deviation; 95% confidence interval. Abbreviations: DOP, dopamine; IL-1, interleukin-1; IL-4, interleukin-4; IL-6, interleukin-6; IL-10, interleukin 10; IL-17, interleukin 17, IL-21, interleukin 21; SER, serotonin; TNF-α, tumor necrosis factor alfa. [1]Data analyzed by *T*-test for independent variables; effect size expressed as Cohen's *d* . [2]Data analyzed Mann-Whitney *U*-test; effect size expressed as Glass's rank-biserial correlation coefficient (r_g).

Supplementary table 3. Changes in hematological indices

Indicator	Units	BOV-COL	PLA	BOV-COL *vs.* PLA *p*-value Cohen's *d* or r_g
WBC	$10^9 \cdot L^{-1}$	(-)0.84 ± 2.40	(-)0.16 ± 2.10	0.252[2]
		((-)2.03 – 0.36)	((-)1.57 – 1.25)	-0.236
LYM	$10^9 \cdot L^{-1}$	(-)0.25 ± 0.45	(-)0.35 ± 0.51	0.577[1]
		((-)0.47 – (-)0.03)	((-)0.70 – (-)0.01)	0.216
MON	$10^9 \cdot L^{-1}$	0.17 ± 0.17	0.24 ± 0.11	0.121[2]
		(0.09 – 0.26)	(0.17 – 0.32)	-0.354
GRA	$10^9 \cdot L^{-1}$	(-)0.77 ± 2.12	((-)0.05 ± 2.01	0.216[2]
		((-)1.83 – 0.28)	((-)1.40 – 1.30)	-0.283
LYM %	p.p.	1.69 ± 6.60	(-)1.93 ± 7.99	0.196[1]
		((-)1.59 – 4.98)	((-)7.29 – 3.44)	0.507
MON %	p.p.	2.97 ± 1.37	2.97 ± 1.16	0.990[1]
		(2.29 – 3.65)	(2.19 – 3.75)	-0.005
GRA %	p.p.	(-)4.66 ± 7.45	(-)1.05 ± 8.55	0.241[1]
		((-)8.37 – (-)0.95)	((-)6.79 – 4.70)	-0.459
RBC	$10^{12} \cdot L^{-1}$	(-)0.02 ± 0.08	(-)0.02 ± 0.08	0.963[1]
		((-)0.05 – 0.02)	((-)0.07 – 0.04)	0.018
HCT	p.p.	(-)0.000 ± 0.017	(-)0.003 ± 0.011	0.685[1]
		((-)0.009 – 0.008)	((-)0.010 – 0.005)	0.157
HGB	$mmol \cdot L^{-1}$	(-)0.53 ± 0.26	(-)0.58 ± 0.17	0.528[1]
		((-)0.66 – (-)0.40)	((-)0.70 – (-)0.47)	0.245
MCV	fL	0.27± 1.21	0.28 ± 1.26	0.975[1]
		((-)0.34 – 0.87)	((-)0.56 – 1.13)	-0.012
MCH	fmol	(-)0.09 ± 0.05	(-)0.10 ± 0.04	0.691[1]
		((-)0.11 – (-)0.06)	((-)0.12 – (-)0.07)	0.154
MCHC	$mmol \cdot L^{-1}$	(-)1.04 ± 0.53	(-)1.16 ± 0.32	0.514[1]
		((-)1.31 – (-)0.78)	((-)1.38 – (-)0.94	0.253
RDW-C	%	0.20 ± 0.77	0.25± 0.72	0.876[1]
		((-)0.18 – 0.58)	((-)0.24 – 0.73)	-0.060
RDW-S	fL	2.31 ± 3.09	2.04 ± 2.38	0.803[1]
		(0.77 – 3.85)	(0.44 – 3.63)	0.096
PLT	$10^9 \cdot L^{-1}$	50 ± 36	59 ± 47	0.551[1]
		(32 – 68)	(28 – 91)	-0.231
MPV	fL	(-)0.07 ± 0.24	(-)0.35 ± 0.44	**0.030[1]**
		((-)0.18 – 0.58)	((-)0.65 – 0.06)	**0.877**
PCT	$cl \cdot L^{-1}$	0.042 ± 0.027	0.045 ± 0.037	0.777[1]
		(0.028 – 0.055)	(0.020 – 0.070)	-0.110
PDW	%	0.11 ± 1.86	(-)0.55 ± 1.29	0.308[1]
		((-)0.81 – 1.04)	((-)1.42 – 0.31)	0.398
PLCR	%	0.18 ± 2.32	(-)1.91 ± 4.34	0.102[1]
		((-)0.98 – 1.33)	((-)4.82 – 1.00)	0.649

Results expressed as means ± standard deviation; 95% confidence interval. Abbreviations: GRA, granulocytes; HCT, hematocrit; HGB, hemoglobin; LYM, lymphocytes; MCH, mean corpuscular hemoglobin mass; MCHC, mean corpuscular hemoglobin concentration; MCV, mean corpuscular volume; MON, monocytes; MPV, mean platelet volume; PCT, platelet hematocrit; PDW, platelet distribution width; PLT, platelet count; PLCR, platelet large cell ratio; RBC, red blood cells; RDW-C, red blood cells distribution width – coefficient of variation; RDW-S, red blood cells distribution width – standard deviation; WBC, white blood cells. [1]Data analyzed by *T*-test for independent variables; effect size expressed as Cohen's *d*. [2]Data analyzed Mann-Whitney *U*-test; effect size expressed as Glass's rank-biserial correlation coefficient (r_g).
